# Allergic Rhinoconjunctivitis: the Role of Histamine

**DOI:** 10.1155/S0962935194000220

**Published:** 1994

**Authors:** M. Andersson, L. Greiff, C. Svensson

**Affiliations:** Department of Otorhinolaryngology University Hospital Lund S-221 85 Sweden

## Abstract

Allergic rhinoconjunctivitis is the most common atopic condition encountered in clinical practice. Analysis of the pathogenesis of this condition permits identification of optimal therapeutic targets. The increased knowledge of the underlying pathophysiology suggests that multiple inflammatory mediators are involved in the pathogenesis of the allergic reaction in the ocular and nasal mucosa. However, despite the presence of a wide range of different mediators, it would appear that histamine plays a key role. Experimental allergen challenge studies have demonstrated that histamine is the only mediator which produces the full spectrum of clinical manifestations of the acute allergic reaction when applied to the mucosal surface. While both H_1_- and H_2_-receptors are present in the nasal and ocular mucosa, only H_1_-receptor antagonists are capable of inhibiting histamine-induced symptoms of allergic rhinoconjunctivitis. Furthermore, although the exact role of histamine in the immediate and prolonged allergic reaction has not yet been fully elucidated, these findings do not exclude the possibility that histamine is involved in these processes. The available evidence therefore supports current clinical practice for use of H_1_-receptor antagonist as a first-line therapy in patients with this atopic condition.


Mediators of Inflammation 3, 171–175 (1994)

### Allergic rhinoconjunctivitis: the role of histamine

M. Andersson,[CA] L. Greiff and C. Svensson

Department of Otorhinolaryngology, University Hospital, S-221 85 Lund, Sweden

[CA]Corresponding Author

**ALLERGIC rhinoconjunctivitis is the most common atopic condition encountered in clinical practice. Analysis of the pathogenesis of this condition permits identification of optimal therapeutic targets. The increased knowledge of the underlying pathophysiology suggests that multiple inflammatory mediators are involved in the pathogenesis of the allergic reaction in the ocular and nasal mucosa. However, despite the presence of a wide range of different mediators, it would appear that histamine plays a key role. Experimental allergen challenge studies have demonstrated that histamine is the only mediator which produces the full spectrum of clinical manifestations of the acute allergic reaction when applied to the mucosal surface. While both $H_1$- and $H_2$-receptors are present in the nasal and ocular mucosa, only $H_1$-receptor antagonists are capable of inhibiting histamine-induced symptoms of allergic rhinoconjunctivitis. Furthermore, although the exact role of histamine in the immediate and prolonged allergic reaction has not yet been fully elucidated, these findings do not exclude the possibility that histamine is involved in these processes. The available evidence therefore supports current clinical practice for use of $H_1$-receptor antagonist as a first-line therapy in patients with this atopic condition.**

**Key words:** Allergic rhinoconjunctivitis, Histamine, Mast cell, Immediate allergic reaction, Airway inflammation

### Allergic rhinitis

Allergic rhinitis is the most common form of allergic disease encountered in clinical practice, afflicting approximately 10–20% of the general population,[1] with the available epidemiological data indicating that the prevalence of this condition is increasing.[2,3] Allergic rhinitis may be seasonal or perennial. Seasonal allergic rhinitis, also known as hayfever, is caused by sensitivity to aeroallergens such as pollens. Triggering allergens for perennial allergic rhinitis are house dust mite, animal dander and mould. Seasonal and perennial allergic rhinitis display similar symptomatology, with both conditions characterised by nasal itching, sneezing, congestion and rhinorrhoea. However, symptoms of perennial allergic rhinitis tend to be less severe, except under conditions of high allergen exposure.

The past decade has witnessed an increasing interest and understanding of the inter-relationship between the upper and lower respiratory tract. In some patients it seems that allergic rhinitis may predispose to the development of asthma,[4] the most serious manifestation of airway allergy,[3,5] suggesting that allergic rhinitis should therefore be regarded as a risk factor for development of generalised airway disease. This is further implied by the common association between allergic rhinitis and bronchial hyperreactivity.[6] Recent data also suggest that asthma symptoms may improve with appropriate treatment of nasal symptoms in patients with concomitant rhinitis and asthma.[7,8]

The nasal mucosa shares several structural similarities with the tracheobronchial mucosa, most notably the respiratory epithelium with its basement membrane and the adjacent submucosa containing microvessels and glands.[9] Perhaps more importantly, a number of functional similarities also exist.[10] Studies of nasal pathology may therefore also serve to illustrate the process of pathophysiological events occurring in the lower airways.

The nose provides excellent opportunities for studies of the functional aspects of the mucosal allergic reaction. The accessibility of the nasal mucosa enables well controlled provocation with various kinds of stimuli and permits the mucosal responses to be equally well monitored by harvesting and analysis of surface liquids, cells and biopsy specimens.

### Anatomical aspects of the nasal mucosa

The nasal airway is covered by a squamous epithelium in the anterior part of the nose and by a ciliated pseudostratified epithelium in the remaining part of

© 1994 Rapid Communications of Oxford Ltd the cavities. The latter type of epithelium consists of at least four different cell types: ciliated columnar cells, non-ciliated columnar cells, goblet cells and basal cells.[9] The epithelial cells rest on a basement membrane and the apical lateral portions are connected with tight junctions. The goblet cells and the serous and seromucous glands contribute to the production of the airway surface liquids which cover the mucosal surface. Further sources are plasma solutes which enter the lumen at mucosal exudation processes, and condensed water recovered from exhaled air.

The nasal mucosa is supplied by sympathetic and parasympathetic nerves, and by sensory nerves which run in the trigeminal nerve. Stimulation of these sensory C-fibres gives rise to nasal itching and sneezing. The presence of neuropeptides has been demonstrated in both the autonomic and sensory nerves using immunohistochemical techniques.[11,12]

Histamine-induced itching and sneezing can be abolished by topical treatment with $H_1$-receptor antagonists, but not with $H_2$-antagonists, indicating the presence and function of $H_1$-receptors in the nasal mucosa.[13]

The human nasal mucosa has a well developed capillary network. This includes a subepithelial layer of capillaries, which partly are of a fenestrated type.[14] Cavernous sinusoids situated in the deepest parts of the lamina propria[15] regulate the mucosal blood content. In the lower part of the lamina propria, arteriovenous shunts allow the blood to bypass the capillary network, and it has been suggested that these shunts may be involved in nasal thermoregulation.[16] Normally, exchange of solutes occurs across the capillary endothelium. However, when the mucosa is inflamed, the endothelium of the postcapillary venules regulates the plasma exudation process.[17] The presence of venous sinusoids, which are very abundant in the nose, has also recently been demonstrated in the bronchi.[18]

### The allergic reaction

The nasal mucosa contains dendritic cells (Langerhans cells) which appear to be capable of presenting aeroallergenic proteins to mucosal immunocompetent cells leading to IgE-production.[19] These IgE antibodies bind to high-affinity receptors on mast cells and basophils in the nasal mucosa, and perhaps also at low affinity receptors on other cells, such as monocytes, eosinophils and platelets.[20–22] On renewed allergen/mucosal contact, the allergen crosslinks two or more IgE molecules on the surface of these cells initiating the production and release of a number of biochemical mediators and other biological substances such as cytokines. The released substances act on local cells and sensory nerve endings leading to increased nasal congestion, watery and proteinic rhinorrhoea, sneezing and nasal itching. These symptoms characterize the immediate allergic reaction and tend to diminish very rapidly.

Symptoms such as increased nasal patency, occasional sneezing and minor nasal secretion may however persist for a longer period of time. The duration and severity of these symptoms appears to be highly individual and may also be related to both the initial dose of allergen and the sensitivity of the atopic individual. A biphasic allergic response has also been documented in some patients characterized by an immediate allergic response followed some hours later by a recurrence of symptoms and a repeated mucosal output of mediators known as a late phase reaction.[23] Functionally, this prolonged allergic inflammatory response is characterized by increased mucosal reactivity to rechallenge with allergen ('specific reactivity') or to 'nonspecific stimuli' such as histamine or methacholine.[24,25] The timing of this increase in nasal reactivity generally corresponds to an influx and activation of inflammatory cells into the superficial part of the nasal mucosa.[26]

This increased responsiveness of hyperreactivity to rechallenge with the specific allergen and other nonspecific stimuli is seen not only during experimental nasal provocation studies (Fig. 1), but also in clinical disease,[27] a phenomenon known as priming.[28] Studies in patients with active allergic rhinitis have shown that symptom severity is greater at the end of the pollen season for a given degree of pollen exposure compared to that experienced earlier in the season.

The pathophysiological events occurring during continuous allergic disease such as natural hayfever are probably much more complicated than any of the above experimentally-induced reactions. It seems likely that an escalating mixture of the immediate response, prolonged symptoms, late phase response and mucosal hyperreactivity to both allergen and nonspecific stimuli contribute to the symptoms seen during natural disease.

### Metachromatic cells

The mast cell is believed to play a central role in the development of the allergic reaction. Activation of these metachromatic cells, triggered by the binding of allergen to IgE, leads to release of granule-associated mediators, including histamine, from preformed cytoplasmic granules and lipid mediators from intracellular lipid bodies,[29,30] as well as to transcription and synthesis of cytokines.[31]

The role of the mast cell in healthy individuals is not clear, but it has been suggested that it may be involved in the regulation of the growth and differentiation of other cells.[32] Our understanding of the

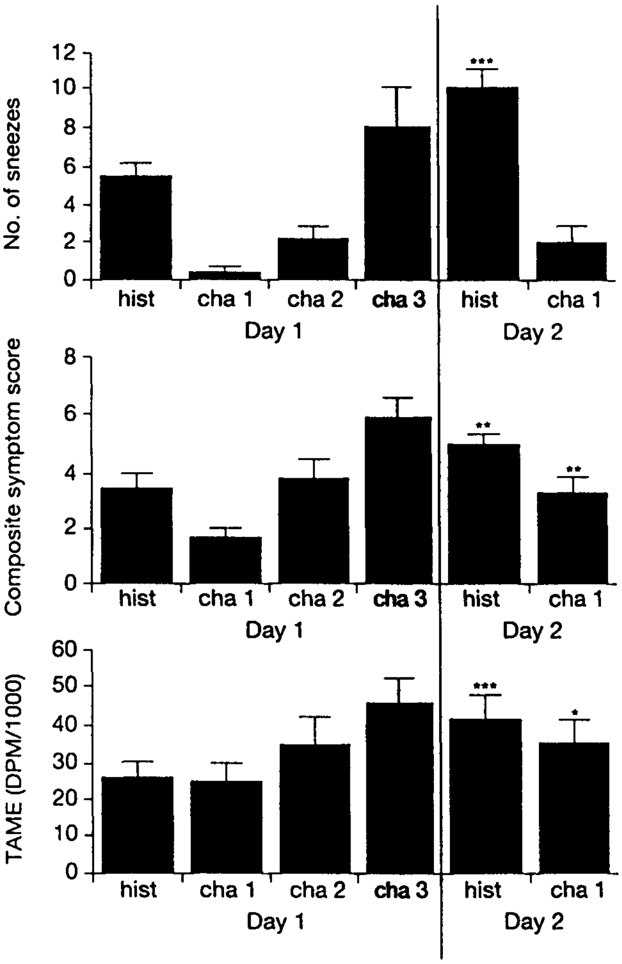

FIG 1. Severity of sneezing (upper panel), composite nasal symptom scores (middle panel) and TAME-esterase levels (lower panel) in nasal lavage fluid after challenge with increasing doses of pollen allergen and histamine (0.1 mg). Patients were rechallenged after 24 h with the lowest dose of allergen and the same dose of histamine. As seen, mucosal reactivity to both allergen and histamine was increased ($n = 13$). *$p < 0.05$, **$p < 0.01$ and ***$p < 0.001$ (day 2 *vs* day 1).

function of this cell is further complicated by findings which would suggest the presence of a specific mucosal mast cell type.[33]

*In vitro* stimulation of the mast cell leads to complex intracytoplasmic changes including the formation of degranulation channels and solubilization of matrix materials prior to the development of multiple openings to the exterior of the degranulation channels.[34] Ultrastructural studies of mast cells have demonstrated that the nasal mucosal mast cells are redistributed from their normal pre-seasonal habitat in the stroma into the epithelium during natural pollen exposure, a phenomenon known as intra-epithelial migration.[35,36] The migratory capacity of the mast cell has also been demonstrated after nasal allergen challenge.[37] In contrast, the migratory capacity of the blood basophil during seasonal disease is controversial. Some authors have reported an increased number of blood basophils during natural pollen exposure,[38] while other studies have failed to confirm these changes.[35,36] However, these discrepancies may partly be due to methodological differences.

Our understanding of the involvement of the mast cell in the pathophysiology of allergic rhinitis has been further derived from the measurement of mediators in nasal lavage fluid. Following allergen challenge in sensitized individuals, increased levels of mediators with a specific mast cell origin such as tryptase and prostaglandin $D_2$ are found in nasal washings.[39–41] Levels of other mediators which may originate from mast cells, as well as from other cells in the nasal mucosa, also increase after local allergen challenge. These include histamine[42] and leukotrienes.[43]

### Histamine

Histamine (β-imidazolethylamine) is an intracellular mediator which exerts a wide range of functions on a variety of target tissues via its effects on $H_1$- and $H_2$-receptors. Produced by decarboxylation of histidine, histamine is widespread in mammalian tissues. Histamine is one of the major mediators of the immediate hypersensitivity reaction.[44–46] Histamine is also an important mediator in the central nervous system and gastric mucosa.[47] Histamine is stored in large amounts in metachromatic granules of mast cells and basophils and is rapidly metabolized by one of two enzymatic pathways, either histamine *N*-methyltransferase or oxidation.[48]

There has been increased interest and research concerning the role of potential mediators such as platelet activating factor, leukotrienes, bradykinins and neurogenic mediators in the pathogenesis of the allergic reaction in recent years. However, histamine is still the only mediator which mimics the full spectrum of nasal symptoms of the immediate allergic response when topically applied to the nasal mucosa. Furthermore, although both $H_1$- and $H_2$-

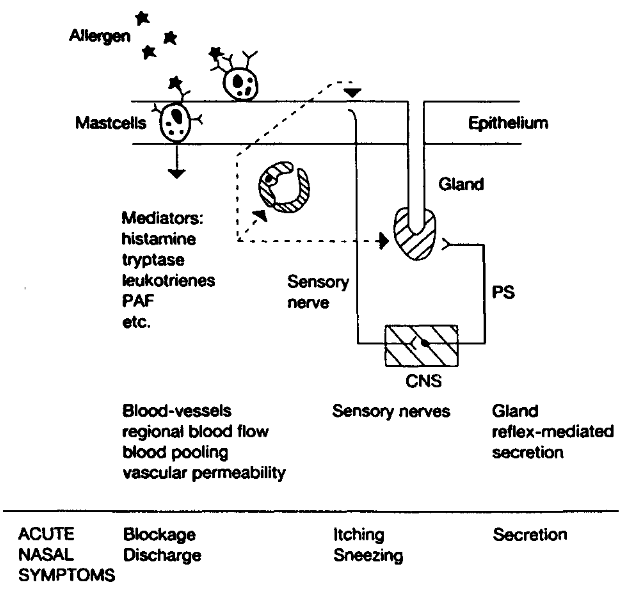

FIG 2. Schematic overview of the effects of histamine on end-organs in the nasal mucosa.

receptors are present in the nasal mucosa, only $H_1$-receptor antagonists are capable of inhibiting this histamine-induced response. As shown in Fig. 2, the effects of histamine in the nose are mediated through binding to receptors on the endothelial cells, vascular smooth muscles and sensory nerves inducing exudation of plasma, vasodilatation, sneezing and nasal itching, respectively.[49] Histamine also induces a reflex-mediated parasympathetic glandular secretion in the nasal mucosa.[50]

Further support for the importance of the mast cell and histamine in the pathogenesis of allergic rhinitis is provided by observations during seasonal disease. Indirect evidence, supported by ultrastructural findings, for mast cell histamine secretory activity has been documented during the pollen season. A strong relationship has been reported between mucosal tissue concentrations of histamine and the severity of nasal symptoms experienced during the pollen season, with a positive correlation between the pre-seasonal number of nasal mast cells and the severity of nasal symptoms during seasonal disease has also been reported.[51]

Although the effects of histamine resemble the effects of allergen in the early allergic reaction, histamine does not, in contrast to allergen, attract and activate eosinophils. Neither does histamine induce increased reactivity of the nasal mucosa to specific or nonspecific stimuli.[52] Furthermore, at present, our knowledge about interactions between histamine and other cells which may be important for the orchestration of allergic airway inflammation, including Langerhans cells, T-lymphocytes and epithelial cells, is limited. However, these findings do not exclude the possibility that histamine is associated with both the immediate and prolonged allergic reaction.

### Allergic conjunctivitis

Allergic conjunctivitis is the most common ocular allergic disorder. Symptoms are typically bilateral, and often simultaneously with allergic rhinitis. However, allergic conjunctivitis can also be present as an isolated manifestation of atopic disease.[53] Conjunctival provocations were performed by Blackley as early as the end of the 19th century.[54] Although ocular experimental allergen challenge studies are performed less frequently than nasal challenges, the available evidence suggests that the pathophysiological mechanisms underlying allergic conjunctivitis and rhinitis are very similar. The pattern of mediators and plasma exudation markers recently observed after allergen challenge of the conjunctiva has previously been demonstrated in allergic rhinitis; consisting of increased levels of kinins, TAME-esterase, albumin and ryptase, indicative of mast cell activation.[55,56] Mast cells have also been identified in conjunctival biopsies from allergic individuals and increased numbers of eosinophils appear in the tear fluid some hours after local allergen challenge.[57] Topical application of histamine to the conjunctiva induces redness and itching, two typical symptoms of allergic conjunctivitis. More pronounced symptoms include swelling of the eye lids which may even develop into a periorbital oedema.[58]

### Implications for patient management

Recent years have witnessed an increasing understanding of the pathophysiology of allergic rhinoconjunctivitis, particularly concerning the potential role of inflammatory mediators, cytokines and inflammatory cells. Histamine appears to play a key role, being the only mediator which induces the full spectrum of clinical manifestations of the immediate allergic reaction when topically applied to the mucosal surface. Coupled with the well-documented efficacy of $H_1$-receptor antagonists in the treatment of allergic rhinoconjunctivitis, these findings provide a pathophysiological rationale for the current clinical practice for use of antihistamines as a therapy in patients with ocular and nasal allergies.

### References

1. Perki JM. Allergy in general practice. *Practitioner* 1972; **208** 776–783.
2. Malmberg H. Symptoms of chronic and allergic rhinitis and occurrence of nasal secretion granulocytes in university students, school children and infants. *Allergy* 1979; **34:** 389–395.
3. Åberg N. Asthma and allergic rhinitis in Swedish conscripts. *Clin Exp Allergy* 1989; **19**: 59–63.
4. Broder I, Higgins MW, Mathews KP, Keller JB. Epidemiology of asthma and allergic rhinitis in a total community. Tecumesh, Michigan. *J Allergy Clin Immunol* 1974; **54**: 100–110.
5. Smith Montgomery J. Knowler LA. Epidemiology of asthma and allergic rhinitis. I. In a rural area. II. In a university-centered community. *Am Rev Respir Dis* 1965; **92**: 16.
6. Cockcroft DW, Killian DN, Mellon JJA, Hargreave FE. Bronchial reactivity to inhaled histamine: a method and clinical survey. *Clin Allergy* 1977; **7**: 235–243.
7. Reed CE, Marcoux MD, Welsh PW. Effects of topical nasal treatment on asthma symptoms. *J Allergy Clin Immunol* 1988; **81**: 1042–1047.
8. Corren J, Aadinoff AD, Buchmeier AD. Nasal beclomethasone prevents the seasonal increase in bronchial responsiveness in patients with allergic rhinitis and asthma. *J Allergy Clin Immunol* 1992; **90**: 250–256.
9. Mygind N. Bisgaard H. Applied anatomy of the airways. In: Mygind N. Pipkorn U, Dahl R, eds *Rhinitis and asthma. Similarities and differences*. Copenhagen, Denmark: Munksgaard, 1990: 21–37.
10. Persson CGA, Svensson C, Greiff L, *et al.* The use of the nose to study the inflammatory response of the respiratory tract. *Thorax* 1992; **47**: 993–1000.
11. Lundberg JM, Terenius L, Hökfelt T, *et al.* Neuropeptide Y (NPY-like immunoreactive in peripheral noradrenergic neurons and effects of NPY on sympathetic function. *Acta Physio Scand* 1982; **116**: 477–480.
12. Udan R, Maim L. Sundler F. Substance P containing nerve fibres in the nasal mucosa. *Arch Otorhinolaryngol* 1983; **238**: 9–16.
13. Secher C, Kirkegaard J., Borum P, Maanson A, Osterhammel P, Mygind N. Significance of $H_1$ and $H_2$ receptors in the human nose: rationale for topical use of combined antihistamine preparations. *J Allergy Clin Immunol* 1982; **70**: 211–218.
14. Cauna N, Hinderer KH. Fine structure of blood vessels of the human nasal respiratory mucosa. *Ann Otol Rhinol Laryngol* 1969; **78** 865–879.
15. Cauna N. Blood and nerve supply of the nasal lining. In: Proctor DF, Andersen IB, eds. *The nose: upper airway physiology and the atmospheric environment*. Amsterdam: Elsevier Biomedical Press, 1982: 45–69.
16. Cole P. Modification of inspired air. In: Proctor DF, Andersen IB, eds. *The nose: upper airway physiology and the atmospheric environment*. Amsterdam: Elsevier Biomedical Press, 1982; 351–375.
17. Grega GJ, Persson CGA, Svensjö E. Endothelial cell reactions to inflammatory mediators assessed by fluid and solute flux analysis. In: Ryan US, Ed. *Endothelial cells*. Boca: CRC Press, 1988: 103–119.
18. Widdicombe JG. Comparison between the vascular beds of upper and lower airways. *Eur Respir J* 1990; **3** (Suppl 12): 564–571.

19. Fokkens WJ, Bruijnzeel-Koomen CAFM, Vroom THM, *et al.* The Langerhans cell: an underestimated cell in atopic disease. *Clin Exp Allergy* 1990; **20**: 627–638.
20. Tada T, Ishizaka K. Distribution of gamma E-forming cells in lymphoid tissues of the human and monkey. *J Immunol* 1970; **104**: 377–387.
21. Grangette C, Gruart V, Ouaissi MA, *et al.* IgE receptor on human eosinophils (FceRH): comparison with B-cell CD23 and association with adhesion molecule. *J Immunol* 1989; **143**: 3580–8.
22. Conrad DH. The receptor for immunoglobulin E. In: Holgate ST, ed. *Mast cells, mediators and disease.* London: Kluwer Academic Publishers, 1988: 99–127.
23. Naclerio RM, Proud D, Togias A, *et al.* Inflammatory mediators in late antigen-induced rhinitis. *N Engl J Med* 1985; **313**: 65–70.
24. Andersson M. Andersson P, Pipkorn U. Allergen-induced specific and nonspecific nasal reactions; reciprocal relationship and inhibition by topical glucocorticosteroids. *Acta Otolaryngol (Stockholm)* 1989; **107**: 270–277.
25. Klementsson H, Svensson C, Andersson M, Venge P, Pipkorn U, Persson CGA. Eosinophils, secretory responsiveness and glucocorticoid-induced effects on the allergic nasal mucosa during a weak pollen season. *Clin Exp Allergy* 1991; **21**: 705–710.
26. Klementsson H, Andersson M, Baumgarten C, Venge P, Pipkorn U. Changes in nonspecific nasal reactivity and eosinophil influx and activation after allergen challenge. *Clin Exp Allergy* 1990; **20**: 539–547.
27. Togias A, Naclerio RM, Proud D, *et al.* Studies on the allergic and nonallergic nasal inflammation. *J Allergy Clin Immunol* 1988; **81**: 782–790.
28. Cornell JT. Quantitative intranasal pollen changes. 3. The priming effect. *J Allergy* 1969; **43**: 33–44.
29. Dvorak AM, Schulman ES, Peters SP, *et al.* Immunoglobulin E-mediated degranulation of isolated human lung mast cells. *Lab Invest* 1985; **53**: 45–56.
30. Dvorak AM, Schleimer RP, Schulman ES, Lichtenstein LM. Human mast cells use conservation and condensation mechanisms during recovery from degranulation. *Lab Invest* 1986; **54**: 663–678.
31. Burd PR, Rogers HW, Gordon JR, *et al.* Interleukin 3-dependent and -independent mast cells stimulated with IgE and antigen express multiple cytokines. *J Exp Med* 1989; **170**: 245–257.
32. Schwartz LB, Huff T. Biology of mast cells and basophils. In: Middleton E Jr, Reed CE, Ellis EF, Adkinson MF Jr, Ynginger JQ, Busse W, eds. *Allergy. Principles and Practice.* 1. St Louis, Missouri: Mosby-Year Book, Inc., 1993: 135–168.
33. Enerbäck L. Mast cell heterogeneity: the evolution of the concept of a specific mucosal mast cell. In: Befus D, Denburg J, Bienenstock J, eds. *Mast cell heterogeneity and derivation.* New York: Raven Press, 1986: 1–26.
34. Dvorak A, Galli SJ, Schulman ES, Lichtenstein LM, Dvorak HF. Basophil and mast cell degranulation: ultrastructural analysis of mechanisms of mediator release. *Fed Proc* 1983; **42**: 2510–2515.
35. Enerbäck L, Pipkorn U, Granerus G. Intraepithelial migration of nasal mucosal mast cells in hayfever. *Int Arch Allergy Appl Immunol* 1986; **80** 44–51.
36. Enerbäck L, Pipkorn U, Olofsson A. Intraepithelial migration of nasal mucosal mast cells in hayfever. Ultrastructural observations. *Int Arch Allergy Appl Immunol* 1986; **81**: 289–297.
37. Juliusson S, Pipkorn U, Karlsson G, Enerbäck L. Mast cells and eosinophils in the allergic mucosal response to allergen challenge: changes in distribution and signs of activation in relation to symptoms. *J Allergy Clin Immunol* 1992; **90**: 898–909.
38. Okuda M, Sakaguchi Y. Suzuki F, Otsuka H, Kawabori S. Ultrastructural heterogeneneity of the basophilic cells in the allergic nasal mucosa. *Ann Allergy* 1985; **54**: 152–157.
39. Naclerio RM, Meter HL, Kagey-Sobotka A, *et al.* Mediator release after nasal airway challenge with allergen. *Am Rev Respir Dis* 1983; **128**: 597–602.
40. Castelle M, Schwartz LB. Tryptase levels in nasal lavage fluid as an indicator of the immediate allergic response. *J Allergy Clin Immunol* 1988; **82**: 348–355.
41. Juliusson S, Holmberg K, Baumgarten C, Olsson M, Irander I, Pipkorn U. Tryptase in nasal lavage fluid after local allergen challenge. Relationship to histamine and TAME-esterase activity. *Allergy* 1991; **46**: 459–465.
42. Andersson M, Nolte H, Olsson M, Stahl Skov P, Pipkorn U. Measurement of histamine in nasal lavage fluid; comparison of a glass fibre based fluorometric method with two radioimmunoassays. *J Allergy Clin Immunol* 1990; **86**: 815–820.
43. Shaw RJ, Fitzharris P, Cromwell O, Wardlaw AJ, Kay AB. Allergen-induced release of sulphopeptide leukotrienes (SRS-A) and LTB4 in allergic rhinnitis. *Allergy* 1985; **40**: 16.
44. Siraganian RP. Histamine secretion from mast cells and basophils. *Trends Pharmcol Sci* 1982; **4**: 432–437.
45. Wassermann SI. Mediators of immediate hypersensitivity. *J Allergy Clin Immunol* 1983; **72**: 101–115.
46. Schwartz LB. Mediators of human mast cells and human mast cell subsets. *Ann Allergy* 1987; **58**: 226–235.
47. Sandvik AK, Waldum HL. Gastrin produces an immediate and dose-dependent histamine release preceeding acid secretion in isolated vasculary perfused rat stomach. *Scand J Gastroenterol* 1987; **22**: 803–808.
48. Holgate ST, Robinson C, Church MK. Mediators of immediate hypersensitivity. In: Middleton E Jr, Reed CE, Ellis EF, Adkinson MF Jr, Ynginger JQ, Busse W, eds. *Allergy. Principle and Practice. I.* St Louis, Missouri: Mosby-Year Book Inc, 1993: 267–301.
49. Svensson C, Baumgarten CR, Pipkorn U, Alkner U, Persson CGA. Reversibility and reproducibility of histamine induced plasma leakage in nasal airways. *Thorax* 1989; **44**: 13–18.
50. Mygind N. Mediators of nasal allergy. *J Allergy Clin Immunol* 1982; **70**: 149–159.
51. Pipkorn U, Karlsson G, Enerbäck L. Secretory activity of nasal mucosal mast cells and histamine release in hayfever. *Int Arch Allergy Appl Immunol* 1988; **87**: 349–360.
52. Grönborg H, Borum P, Mygind N. Histamine and methacholine do not increase nasal reactivity. *Clin Allergy* 1986; **16**: 597–602.
53. Marrache F, Brunet D, Frandeboeuf J, *et al.* The role of ocular manifestations in childhood allergy syndromes. *Rev Fr Allergol Immunol Clin* 1978; **18**: 151–155.
54. Blackley CH, *Experimental researches on the causes and nature of catarrheus aestivus.* Ballier Tindal and Cox, London 1873.
55. Wenzel S, Irani AA, Sanders JM *et al.* Immunoassay of tryptase from human mast cells. *Immunol Method* 1986; **86**: 139–142.
56. Proud D, Sweet J, Stein P, *et al.* Inflammatory mediator release of conjunctival provocation of allergic subjects with allergen. *J Allergy Clin Immunol* 1990; **85**: 896–905.
57. Bonini S, Tracine SD, Barney NP, *et al.* Late phase reaction and tear fluid cytology in the rat ocular anaphylaxis. *Curr Eye Res* 1987; **6**: 659–665.
58. Bielory L, Frohman LP. Allergy and immunologic disorders of the eye. *J Allergy Clin Immunol* 1992; **89**: 1–17.

ACKNOWLEDGEMENTS This research was supported in part by grants from the Swedish Medical Research Council (project no. 8308), the Swedish Association against Asthma and Allergy, and the Medical Faculty at the University of Lund.

**Received 12 January 1994; accepted 2 February 1994**
